# ‘We are all in this together’: Investigating alignments in intersectoral partnerships dedicated to K-12 food literacy education

**DOI:** 10.1177/00178969211011522

**Published:** 2021-04-23

**Authors:** Kerry Renwick, Lisa Jordan Powell, Gabrielle Edwards

**Affiliations:** a Department of Curriculum & Pedagogy, Faculty of Education, The University of British Columbia, Vancouver, BC, Canada; b Center for Human & Environmental Sustainability and STEM Division, Sweet Briar College, Sweet Briar, VA, USA

**Keywords:** Food literacy education, food studies curriculum, intersectoral partnerships

## Abstract

**Background:**

Activities to foster food literacy in young people are increasingly common in schools, driven both by the public health sector and by curriculum mandates from education officials in government. In Canada, both Kindergarten–Grade 12 (K-12) classroom teachers and educators from community organisations deliver food literacy education programmes in schools, often framed as partnerships working in the interests of young people.

**Objective:**

The study examines the alignment between what both classroom teachers and community educators state are the desired outcomes for students of their food literacy education work and the topics/activities they engage in with students.

**Design, setting and method:**

We surveyed and interviewed teachers and community educators in British Columbia, Canada, and utilised participant observation and secondary data from food literacy education network activities.

**Results:**

Shared food literacy education goals and topics/activities were evident in the responses of classroom teachers and community educators. Teachers framed their food literacy education programmes around the curriculum-as-plan – in this case, the provincial curriculum known as the BC Curriculum – and then enacted a lived curriculum that students experienced in the classroom. Community educators offered programmes that were initially designed to meet their organisation’s focus, but which varied in terms of how much of the BC Curriculum was addressed.

**Conclusion:**

Our results show broad alignment between teachers and community educators in food literacy education goals and practices; however, there is potential to increase this alignment and build stronger partnerships that support teachers in enacting the BC curriculum and meeting the needs of their students.

The concept of ‘food literacy’ has garnered increased attention among health educators and other health promotion professionals in recent decades, predicated on the link between dietary patterns and chronic disease prevention (Krause et al., 2018; Ronto et al., 2016). Particular interest has been shown in young people developing food literacy skills and knowledge (Ronto et al., 2016; Slater et al., 2018; Truman et al., 2020), focused on changing behaviours early in life to ‘future-proof’ them for diet-related illness, although they live in complex food environments that support an energy-dense food supply. Several definitions of food literacy have been developed, with most emphasising food preparation skills and individual decision-making (Krause et al., 2018) and little attention given to the social construction of food literacy through family and community contexts or understanding sustainable and socially just food systems (Renwick and Powell, 2019).

Kindergarten–Grade 12 (K-12) schools have experienced a growing number of activities and programmes incorporated under the umbrella of ‘food literacy education’. These include school gardens, classroom cooking, cookbook creation, composting and using food to engage with Indigenous knowledges and understandings. This article examines the goals and desired outcomes of educators who share food literacy education activities with students, including both K-12 classroom teachers and staff members from community non-profit organisations who partner with K-12 schools. Our research sought to identify alignments between goals and practices of K-12 classroom teachers and community educators and how the aims and practices of these groups are conveyed through curriculum that is both intended and experienced.

In particular, the research examines relationships between what community non-profit groups who deliver food literacy education in schools are providing and what classroom teachers engage with as part of their own practice and provincial curriculum mandates. Both of these provide not only content but also contexts for student learning. Aoki (1993) argues that there is more to curriculum than development and instruction. He invites us to also consider the experience that teachers and students have within the classroom context, in terms of what he calls ‘curricular landscapes’ (p. 255). Aoki asks us to think about curriculum-as-plan and the lived curriculum. A curriculum-as-plan is generally developed outside the classroom by curriculum developers as government agents and represents ‘the planners’ orientations to the world’ (Aoki, 1993: 258). In a classroom, the curriculum ‘comes alive’ because of the teacher’s intentions and interactions with their students both individually and as a group. While the teacher may be guided by the planned curriculum, they are also attuned to the needs of individual students and how their learning experiences generate a lived curriculum. With this nomenclature in mind, our research considers the alignment between the objectives of community educators and their organisations and those of teachers and K-12 schools and how programmatic intentions can support teachers and ensure K-12 students to have meaningful lived curriculum experiences.

## Schools as sites for food-related activity

Schools have a long history of being sites for public health interventions. Some are just-in-time annual or age-specific programmes that include dental health, tuberculosis screening, lice or immunisation programmes for German measles, chicken pox, hepatitis B and HPV (human papillomavirus) (Herlitz et al., 2020; Salam et al., 2016). These programmes are usually run by health professionals with a public health goal and little to no presence within the teaching and learning context. Health promotion makes considerable use of school-based interventions categorised as health education and intended to create changes in behaviour. The focus of these interventions is varied and includes mental health, suicide prevention and substance abuse. While some interventions are evident in primary education, they become more visible in secondary education as young people move from childhood into young adulthood and are more likely to engage in rebellious and risk behaviour (Shoveller and Johnson, 2006).

Food-related health promotion interventions are also present in schools. Often, these programmes have been designed by public health nutrition professionals with a specific goal in mind. They are rarely designed in partnership with professional educators or tailored to the school and its community (Rossi and Kirk, 2020). These programmes frequently target health concerns such as obesity, the availability of nutritious food choices, eating behaviour, dietary guidelines (da Silveira et al., 2013; Nguyen et al., 2017; Panunzio et al., 2007) and lunch programmes (Gaddis and Coplen, 2018). With a concern for disease prevention, intervening by commandeering school curricula is a typical approach (Leahy et al., 2015).

Leahy et al. (2015) have noted a proliferation of organisations and corporations that are both developing and implementing health education programmes across regions including North America, Europe and Southeast Asia. Some of these programmes are government-funded; however, non-governmental, community educators also actively seek curriculum space in K-12 schools. While community educators do not necessarily have health promotion as their focus *per se*, they argue that they are contributing to the health and wellbeing of young people and the broader community and educating about sustainability issues. Within food and nutrition education, there are a number of approaches to thinking about interventions to support health concerns about young people and their eating.

To access K-12 schools, intervention programmes are justified on the basis of their work being what Leahy et al. (2015) call an ‘undeniable good’ (p. 103) and where the school community is positioned to work for health gains given that ‘we are all in this together’ (p. 105). Intersectoral partnerships have been a foundational principle in health promotion (World Health Organization [WHO], 1986). While Dubois et al. (2015) have ‘noted the lack of a comprehensive, explanatory conceptual model for intersectoral process’ (p. 2940), the belief that intersectoral partnerships remain relevant and central in a health promotion strategy has rarely wavered (Corbin et al., 2018). The commandeering of the classroom, young people, their families and broader school community by a health promotion agency or community educator is framed as collaborative action and partnership in the interests of young people. Key attributes of the partnership include shared mission and the strategic use of resources that include diverse participation, communication, diffused leadership, communication and evaluation (Corbin et al., 2018).

The use of community education programmes by schools is an example of what Rossi and Kirk (2020) describe as outsourcing. The effectiveness of outsourcing relates in part to the differences between Aoki’s (1993) curriculum-as-plan and the lived curriculum. Each of Aoki’s descriptions of the curriculum requires being alert to the tension between curriculum fidelity and innovation (Rossi and Kirk, 2020). Fidelity concerns how closely a teacher implements the planned curriculum, and innovation is evident in how a teacher adjusts and varies the planned curriculum to meet the needs of students in the classroom. It is the difference between Aoki’s two curricula – curriculum-as-plan and the lived curriculum – that highlights the need for community educators and teachers to work in partnership.

## Our framing of food literacy

The focus of what is claimed as food literacy practice is usually dependent upon the intent behind the offered experiences. Renwick and Smith (2020) note four discourses that pervade the justification of food literacy – the prevalence of nutritionism, the loss of cooking skills, unsustainable eating practices, and concern for food justice. These discourses underscore the integrated and cross-disciplinary nature of food and how our relationship with food is not only situated within understandings of our bodies ‘but also in complex food systems and that individual decisions influence many spheres beyond the health of one’s own body’ (Renwick and Powell, 2019: 48).

Food literacy includes knowledges that are created in, and because of, our interactions with socio-cultural, socio-political and environmental contexts (Renwick and Powell, 2019). Cullen et al. (2015) have argued that food literacy is

the ability of an individual to understand food in a way that they develop a positive relationship with it, including food skills and practices across the lifespan in order to navigate, engage and participate within a complex food system. Food literacy is the ability to make decisions to support the achievement of personal health and a sustainable food system considering environmental, social, economic, cultural and political components. (p. 143)

Cullen et al.’s (2015) definition acknowledges human activity around food in personal and community contexts as well as the need for sustainable food systems; our own work aligns with this holistic view. Informed by Green’s (2012) three-dimensional model of literacy, we sought to understand how teachers and community educators conceptualise and provide food literacy activities according to operational, cultural and critical literacy dimensions (Renwick, 2017). Each of these dimensions has a specific intent to ‘read’ the world in a particular way. The operational dimension focuses on ‘making things work’, such as being able to grow food, knowing where food comes from and being able to read recipes and prepare food/meals. The cultural dimension concerns what is meaningful and effective so that we can understand family choices, marketing and product loyalty, and diverse social environments. Critical literacy is the dimension by which an understanding of systems and power relations comes to the fore and is evident in engaging with political action, food policy and food justice.

Very little is known about how food literacy education operates in the classroom (Brooks and Begley, 2014). In British Columbia, the Ministry of Education’s (2018a) Curriculum includes specific content in the Applied Design, Skills and Technologies (ADST) subject area related to food literacy and what Aoki refers to as the curriculum-as-plan. Teachers also determine both what and how curriculum content is delivered to young people. They may assume responsibility for teaching food literacy themselves or choose from the food literacy programmes offered by non-profit organisations or other service providers that come into schools or invite classes to visit their facilities. Therefore, it is important to explore how teachers and programme providers understand food literacy and incorporate related ideas and activities into their practice. This is what constitutes the lived curriculum experience. Furthermore, as food literacy education efforts increase in schools, there is a need to understand the capacities they are building and how these relate to efforts to transform food systems to be more socially just and environmentally sustainable.

## Setting

The research took place in Vancouver, British Columbia, with additional work in other parts of the province. Vancouver has an international reputation as an urban area focused on sustainability, supported by the city’s plan to become ‘the greenest city in the world’ (City of Vancouver, 2015). Vancouver is home to multiple local, regional and provincial groups dedicated to increased environmental sustainability and social justice in food systems. Numerous non-profit organisations work in the city on educational programmes for children and youth about aspects of food literacy. These include groups focusing on classroom cooking and others focusing on gardening in school grounds. Most Vancouver School Board (VSB) schools have a school garden, and the VSB has a school garden policy (VSB, 2010). Engaging with food literacy reflects the espoused intent of the BC Curriculum to enable the province’s students ‘to foster deeper, more transferable learning’ in their relationships, the natural environment and the contributions they make to their communities (Ministry of Education, 2018a). Members of the project’s research team have been involved with advisory and working groups through the Vancouver School Food Network (VSFN). These groups include representatives from non-profit organisations, VSB, Vancouver Coastal Health and other interested community members who meet to share information and discuss issues related to school food and food literacy education.

Our research engaged substantially with a separately funded community–university educational programming partnership, Think&EatGreen@School 2017-2019 (TEGS). This project built on the foundations set by an earlier phase of the programme, funded from 2010 to 2015 (Rojas et al., 2017). TEGS was a partnership between the City of Vancouver, VSB and the University of British Columbia (UBC) Centre for Sustainable Food Systems, offering small grants to elementary and secondary schools and community organisations within the City of Vancouver to implement school food systems projects. TEGS also hosted a summer institute for teachers and co-hosted food literacy–related professional development events. While the primary role of TEGS was not to deliver food literacy education, it provided resources and events to those who did. Educators involved in TEGS grant–supported activities formed a network of individuals within the VSB such as teachers, administrators and Parental Advisory Committees, and outside the VSB, including community organisations with long-standing relationships with schools and teachers. These stakeholders expressed a common interest in school food systems and food literacy education, although their individual experiences varied widely. The TEGS network provided an initial pool of potential participants in research activities. The reporting structure for TEGS-funded projects also provided a source of secondary data to use for the project.^1^

## Method

This study employed multiple qualitative and ethnographic methods, including surveys, interviews, participant observation and the use of secondary data. As part of a larger research project, through surveys and interviews we sought to gain information on how individuals providing food literacy education programming frame and inform their work, what they see as their objectives and desired outcomes, and what they actually do with students in practice – that is, how educators implemented the planned curriculum and what they developed as a lived curriculum. Through our engagement with the VSFN and participants in TEGS programmes, we also learned how food systems educators framed their work to their peers (both within and across categories of classroom teachers and community educators). Secondary data, in the form of applications and reports submitted to the TEGS programme, provided documentation of how educators framed their objectives and what activities they did under the label of ‘food literacy education’.

### Survey

We developed a survey for both K-12 classroom teachers and others who provide food literacy education, including educators from community organisations and government agencies. To develop the survey, we conducted multiple rounds of question-generation exercises, both within our research team and with members of the VSFN, to ensure our questions would generate information of use to the food literacy educator community. The study team refined the list of possible questions and developed an initial test survey using Qualtrics, the survey platform provided by our university. Three members of the Vancouver school food education community tested the survey and provided feedback on its usability and clarity. We made slight adjustments to the survey based on their feedback. The survey included questions about educator demographics, food literacy topics taught, expected learning outcomes of food literacy education activities, educators’ motivations for food literacy education and resources used. Questions took the format of Likert-type scales, rankings, and multiple-choice and open-ended questions. In this article, we analyse results of two of the survey questions that focus on what educators see as their desired learning outcomes and the types of activities they offer young people.

We launched the survey in February 2019, initially distributing it by email to teachers and community educators who were current members of the TEGS network and VSFN. We sought snowball distribution by asking those who initially received the survey to share it with any other educators who engaged in food systems education activities and would be potential respondents. Following this round of distribution within Vancouver, we expanded distribution to include members of Teachers of Home Economics Specialist Association (THESA), which works across the province of British Columbia. When we closed the survey in June 2020, there were a total of 49 respondents across all distributions.

At the end of the online survey, respondents had the option of indicating whether they were willing to be contacted for a follow-up interview. The initial brainstorming sessions for developing survey questions had resulted in a list of questions more appropriately left for interviews, and our research team drew on this to begin developing our interview questions. We also developed questions based on themes that had emerged in the open-ended survey questions. Sixteen interviews were conducted between March 2020 and August 2020. These interviews were fully transcribed and analysed for common themes.

### Participant observation

The research team attended VSFN and THESA meetings as members of the networks. During these meetings, we listened to and participated in conversations about the activities of different food literacy education practitioners and stakeholders, and the concerns and goals they had. Members of the team also served as facilitators of TEGS events, including professional development workshops and a Summer Institute held in both Summer 2018 and 2019. These events included hands-on training sessions in gardening and cooking, as well as talks on topics such as food justice and food sovereignty, followed by Q&A sessions. Conversations and observations during these events informed this research.

### Secondary data

The research also relied on secondary data accessed in project applications and reports collected through the TEGS project activities.

## Results

The BC Curriculum provides provincial teachers with curricular frameworks that are ‘described as concept-based and competency-driven’ (Ministry of Education, 2018a: n.p.). The BC curriculum is what Aoki (1993) calls the curriculum-as-plan that originates outside the classroom from government authority. While not explicit in the BC Curriculum, directives for food literacy education are implicit in the curriculum areas of ADST, Physical and Health Education (PHE) and Science education. Classroom teachers must work within the frameworks of the BC Curriculum; hence, its content and approach are evident in their food literacy education goals and practices.

Among the multiple community organisations in Vancouver working with schools to deliver food literacy education, some are solely dedicated to food-focused work, while others (e.g. community centres and neighbourhood houses) include food systems education work as part of a broader suite of services. TEGS provided small grants (up to US$2000) for which these organisations could apply; in 2018–2019, nine organisations applied for these small grants. Five of these organisations had missions that primarily focus on food systems education; two were organisations that focused on environmental education with food systems education as a key aspect of this; and for two, food-related programming was one area among many community-building and social support activities. Only one of the organisations could be categorised as a public health organisation, although seven of the nine organisations included information about how they support healthy eating and/or nutrition through their educational programmes. Among the nine organisations, one provided a mission/vision statement that explicitly included integrating their educational efforts with the BC curriculum; however, two additional groups stated that the activities they conducted in schools were designed to integrate with the BC curriculum. Three of the organisations thus acknowledged the value of the BC Curriculum, whereas the remaining six were drawing on their organisation’s goals to drive their curriculum-as-plan.

**Figure 1.**
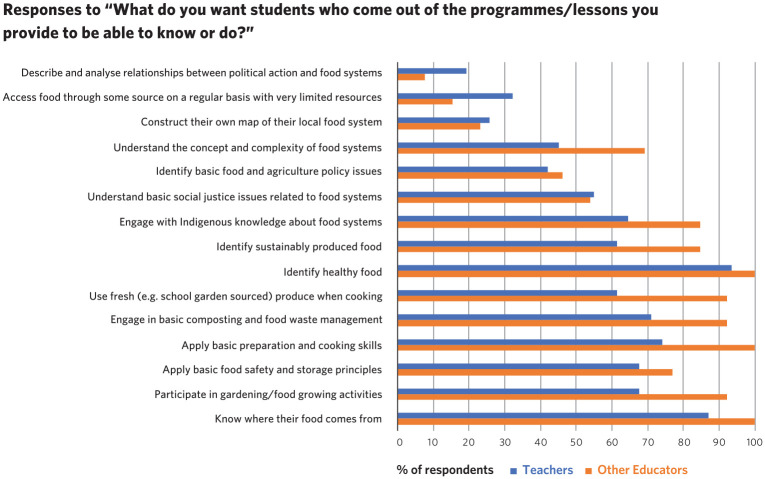
Food literacy educators’ desired outcomes for students.

Survey and interview questions sought to understand the desired outcomes/goals of food literacy educators: that is, what they wanted students to gain from the curriculum they provided. Through responses to the survey question ‘What do you want students who come out of the programmes/lessons you provide to be able to know or do?’ K-12 and community educators identified the goals they had in working with students. Figure 1 details answers from both K-12 classroom teachers and community educators; participants were instructed to select all the options that applied.

As can be seen in Figure 1, high percentages (over 70%) of both classroom teacher and community educator respondents had desired outcomes of students knowing where food comes from, applying basic food preparation and cooking skills, engaging in composting and waste management, and identifying healthy food. All of these goals fit within the operational component of food literacy. Under 30% of respondents from both groups stated their goals for students included being able to construct a map of their local food system and being able to describe and analyse relationship between political action and food systems. The former lies at the border of cultural and critical food literacy, and the latter is firmly a critical food literacy competency.

The three most selected desired outcomes by teacher survey respondents were the same as the three most selected desired outcomes by community educators: knowing where food comes from, identifying healthy food, and applying basic preparation and cooking skills. In interview, one elementary school teacher noted that ‘If our children of today don’t understand the complexities of what they’re consuming, they’re not going to make good decisions when they actually have their own money’. The ADST curriculum specifically engages with food as a medium at the beginning of the middle years and expands in content and process through to Year 12. One part of this content includes exploring food systems, food policy and sustainability. As stated by a secondary teacher in an interview, ‘. . . it is important to develop their skills and acquire the skills they need to navigate their food system . . .’.

The three least selected desired outcomes by the two groups were also the same: constructing their own map of their local food system, accessing food through some source on a regular basis with very limited resources, and describing and analysing relationships between political action and food systems. While under 20% of respondents selected from both groups described and analysed the relationships between political action and food systems, over twice as many classroom teachers selected this option. The composition of our teacher sample (only 14 secondary teachers compared to 18 elementary teachers) may partially explain why so few teachers selected this option.

In contrast, in an interview, one secondary teacher stated, ‘It’s citizenship, the philosophy in my classroom that supersedes academics. The academics will come but this is creating global citizens, that’s why I’m a teacher’. While this desired outcome was selected by a relatively small percentage of both categories of respondents, the number of classroom teacher respondents (32.26%) who selected the outcome of ‘Access food through some source on a regular basis with very limited resources’ was twice that of community educators (15.38 %). This difference may be due to classroom teachers’ daily interaction with students in the context of their lived curriculum and observing that some were food insecure.

**Figure 2.**
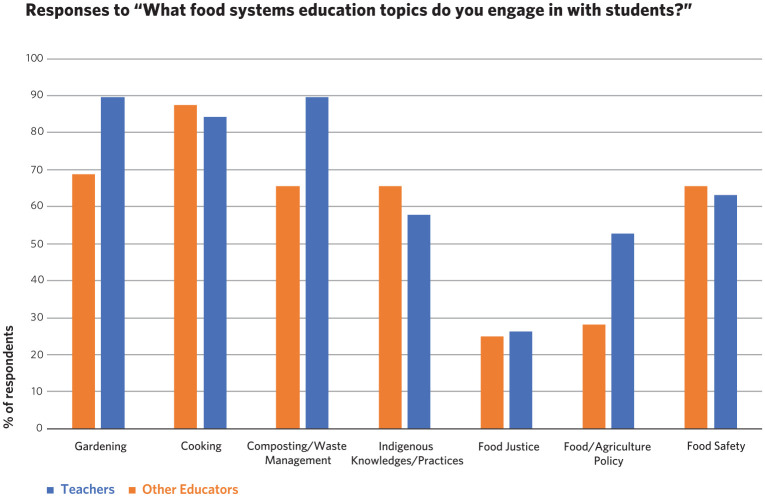
Topics and activities food systems educators engage in with students.

Through the question ‘What food systems education topics do you engage in with students?’ we sought information about what classroom teachers and community educators actually do in practice. Figure 2 summarises the responses of both classroom teachers and community educators.

Comparing the focus on topics by all teachers to that of community educators (see Figure 2), there were some commonalities, but with varied emphasis. A high percentage (over 80%) of both classroom teachers and community educator respondents said they engaged in cooking activities with students. The top five activities that community educator respondents selected aligned with the top five activities selected by classroom teachers (cooking, gardening, composting/waste management, Indigenous knowledge/practices and food safety) – here, we reference five of them because there was a three-way tie in the third most selected option by classroom teachers. Part of the alignment in activities may be due to both groups working with the same students, that is, both teachers and the community educators who come into their classes may report being engaged in an activity with students.

There was an over 20% difference between teachers and community educators in how many respondents from each group said they engaged in educational activities around gardening, composting/waste management and food/agriculture policy with students. In each case, more community educator respondents (nearly 90% for the first two, just over 50% for the last) said they did these activities than teachers. These differences can largely be explained by the aims of the community educators and the organisations that they worked for. One community educator pointed out in interview that ‘Our main goals are supporting food literacy and skills, basic food access and policy advocacy type work’, and others were focused on ‘connect(ing) children to their food, to each other, to nature and to their community’. Another stated they ‘facilitate outdoor experiential learning with teachers and students, mentor youth leaders through garden clubs’. Thus, creating and working in school gardens and composting were significant foci, and these were in part linked to cooking activities, youth development and advocacy work.

## Discussion

When community educators come into schools, the encounters between them and teachers and students create a lived curriculum that provides a variation on the usual classroom experience. While teachers and community educators work to provide educational experiences for young people, what teachers emphasise and implement is influenced by the BC Curriculum, while community educators typically reference their organisation’s mission statement. This difference provides a basis for re-consideration of what else is needed to build effective partnerships around food systems education (Corbin et al., 2018).

When classroom teachers and community educators responded to the question ‘What do you want students who come out of the programmes/lessons you provide to be able to know or do?’ they spoke primarily to the goals established through the planned curriculum. For classroom teachers, the BC Curriculum establishes the planned curriculum, but it does not explicitly frame specific food literacy goals; rather, it includes goals or competencies that can be achieved through food literacy education. While some community educators may be affiliated with one of the few community groups that explicitly frames its work in terms of the BC Curriculum, most are not, and accordingly their planned curriculum originates primarily from the mission of their organisation.

By asking participants in this study to identify ‘What food systems education topics do you engage in with students?’ we were not only looking for alignment between classroom teachers and community educators, but also for alignment between stated goals and what students actually experience in the classroom. The topics most often selected by teachers, cooking and gardening, align with the three most selected goals of teacher respondents – to enable students to identify healthy food, apply basic cooking and preparation skills and know where their food comes from. The focus of the programmes and actual learning activities reflects the decisions that teachers make to create a lived curriculum and how this learning is best facilitated for their students. Areas of commonality for teachers centred on healthy eating, food sources and preparation. These topics largely reflect public health nutrition calls to address diet-related disease (see, for example, Saxe-Custack et al., 2020; Utter et al., 2017). The focus is on the skills development and behaviours associated with operational food literacy, such as ‘How do I select healthy food?’ and ‘How do I cook to manage nutritional value?’ Indigenous knowledge concerning food systems was identified as a desired outcome by more than half of the classroom teacher and community educator respondents. While community educators saw it as a desired outcome, teachers were more likely to engage with students in relation to Indigenous knowledges/practices. The difference between the two group may in part be due to teachers being guided by the rationale in the BC Curriculum for Indigenous knowledge whereby Indigenous perspectives are expected to be integrated in all areas of learning (Ministry of Education, 2018b). Work in this area relates to the cultural food literacy dimension, as food is typically part of everyday experiences so that it can be perceived as a pathway to incorporating Indigenous knowledge and perspectives in classrooms.

Reviewing Corbin et al.’s (2018) key attributes of an effective partnership enables an analysis of the work of teachers and community educators and its inclusivity of partnership practices. While all the attributes of an effective partnership were not evident in this study, it was possible to identify three of the attributes. A shared mission for food literacy education was evident between teachers and community educators, reflecting Aoki’s (1993) curriculum-as-plan. There was also agreement about the majority of concepts and processes that they wanted young people to be able to achieve. The fact that community educators were invited into schools to deliver their programme to young people provides evidence of some degree of diversity in teaching and learning opportunities. The use of resources such as school gardens in strategic ways offered some place-based learning as lived curriculum (Aoki, 1993). However, not all schools or teachers worked with the community educators, and therefore engagement with their programmes varied.

The types of experiences that teacher and community educators provided were broadly similar in that there was a strong focus on operational food literacy; however, the emphasis that particular content was given was dependent on how well the mission of the community educators’ organisation aligned with the BC Curriculum. In those instances where there was not a clearly articulated focus on the BC curriculum, it becomes more difficult for teachers to be able to both justify and supplement once the community educator had left. Attention to fidelity with respect to the BC Curriculum needs to be balanced against the potential for bespoke innovations (Rossi and Kirk, 2020) that is done in partnership with teachers who have the most knowledge about the young people as recipients of the lived curriculum.

## Conclusion

This study examined the alignment between the desired outcomes and topics covered with students by both K-12 classroom teachers and community educators who provide food literacy education in Vancouver and other areas of British Columbia. Our work combined frameworks on intersectoral health promotion partnership and planned and lived curricula to better understand the dynamics of partnerships established in pursuit of food literacy education. Study results indicate that while there was substantial alignment between the two groups, there exists the potential to develop stronger partnerships between classroom teachers and community educators to support teachers working with the BC Curriculum and enhance the practices of both groups in building a lived curriculum for students.

The BC Curriculum as a provincial policy document includes explicit reference to food literacy education, and teachers demonstrated engagement with food literacy education in their programmes. As this is a relatively new area of focus for many, there is potential to build the capacity of teachers to deliver content and integrate it with their pedagogical practices, especially through home economics–focused teacher education, which is currently available in BC teacher education at the secondary but not the elementary level. The tightly focused work of community educators is reflective of their organisations’ mission statements. There is an opportunity, however, for community educators working in school contexts and with teachers to more explicitly reference the BC Curriculum and incorporate insights from teachers about the needs and wants of young people in class.

As we continue to work in this area, we will be looking more at the characteristics of effective partnerships specifically oriented to food literacy education. We will also be exploring how different types of partnerships meet the needs of multiple stakeholders, including students, classroom teachers, community educators and other community members. We are also considering how different types of partnerships can support education across the operational, cultural and critical components of food literacy.

## Notes

1. The programme ceased operating in mid-2019. However, undergraduate students in University of British Columbia’s Faculty of Land and Food Systems continue to work closely with Vancouver teachers and community groups, and the UBC campus farm continues to host food literacy–focused professional development activities for educators.

## References

[bibr1-00178969211011522] Aoki TT (1993) Legitimating lived curriculum: Towards a curricular landscape of multiplicity. Journal of Curriculum and Supervision 8(2): 255.

[bibr2-00178969211011522] Brooks N Begley A (2014) Adolescent food literacy programmes: A review of the literature. Nutrition & Dietetics 71(3): 158–171.

[bibr3-00178969211011522] City of Vancouver (2015) Greenest city 2020 action plan. Report, City of Vancouver, Vancouver, BC, Canada.

[bibr4-00178969211011522] Corbin J Jones J Barry MM (2018) What makes intersectoral partnerships for health promotion work? A review of the international literature. Health Promotion International 33(1): 4–26. 27506627 10.1093/heapro/daw061 PMC5914378

[bibr5-00178969211011522] Cullen T Hatch J Martin W, et al. (2015) Food literacy: Definition and framework for action. Canadian Journal of Dietetic Practice and Research 76(3): 1–6. 26280794 10.3148/cjdpr-2015-010

[bibr6-00178969211011522] da Silveira JAC Taddei JA de AC, et al. (2013) The effect of participation in school-based nutrition education interventions on body mass index: A meta-analysis of randomized controlled community trials. Preventive Medicine 56(3–4): 237–243. 23370048 10.1016/j.ypmed.2013.01.011

[bibr7-00178969211011522] Dubois A St-Pierre L Veras M (2015) A scoping review of definitions and frameworks of intersectoral action. Ciencia and Saude Coletiva 20(10): 2933–2942. 26465838 10.1590/1413-812320152010.01222014

[bibr8-00178969211011522] Gaddis J Coplen AK (2018) Reorganizing school lunch for a more just and sustainable food system in the US. Feminist Economics 24(3): 89–112.

[bibr9-00178969211011522] Green B (2012) Subject-specific literacy and school learning: A revised account. In: Green B Beavis C (eds) Literacy in 3D: An Integrated Perspective in Theory and Practice. Melbourne, VIC, Australia: Australian Council for Educational Research, pp. 2–22.

[bibr10-00178969211011522] Herlitz L MacIntyre H Osborn T, et al. (2020) The sustainability of public health interventions in schools: A systematic review. Implementation Science 15(1): 1–28. 31906983 10.1186/s13012-019-0961-8 PMC6945701

[bibr11-00178969211011522] Krause C Sommerhalder K Beer-Borst S, et al. (2018) Just a subtle difference? Findings from a systematic review on definitions of nutrition literacy and food literacy. Health Promotion International 33(3): 378–389. 27803197 10.1093/heapro/daw084 PMC6005107

[bibr12-00178969211011522] Leahy D Burrows L McCuaig L, et al. (2015) School Health Education in Changing Times: Curriculum, Pedagogies and Partnerships. Oxon: Routledge.

[bibr13-00178969211011522] Ministry of Education (2018a) Curriculum orientation guide. Available at: https://curriculum.gov.bc.ca/sites/curriculum.gov.bc.ca/files/Curriculum_Brochure.pdf (accessed 9 August 2020).

[bibr14-00178969211011522] Ministry of Education (2018b) Indigenous knowledge and perspectives in K-12 curriculum. Available at: https://curriculum.gov.bc.ca/curriculum/indigenous-education-resources/indigenous-knowledge-and-perspectives-k-12-curriculum (accessed 9 August 2020).

[bibr15-00178969211011522] Nguyen KA de Villiers A Fourie JM, et al. (2017) Challenges to implementing the food-based dietary guidelines in the South African primary school curriculum: A qualitative study exploring the perceptions of principals and curriculum advisors. South African Journal of Clinical Nutrition 30(1): 15–20.

[bibr16-00178969211011522] Panunzio MF Antoniciello A Pisano A, et al. (2007) Nutrition education intervention by teachers may promote fruit and vegetable consumption in Italian students. Nutrition Research 27(9): 524–528.

[bibr17-00178969211011522] Renwick K (2017) Critical health literacy in 3D. Frontiers in Education 2: 40.

[bibr18-00178969211011522] Renwick K Powell LJ (2019) Focusing on the literacy in food literacy: Practice, community, and food sovereignty. Journal of Family and Consumer Sciences 111(1): 24–31.

[bibr19-00178969211011522] Renwick K Smith MG (2020) The political action of food literacy: A scoping review. Journal of Family and Consumer Sciences 112(1): 14–22.

[bibr20-00178969211011522] Rojas A Black J Orrego E, et al. (2017) Insights from the Think&EatGreen@School Project: How a community-based action research project contributed to healthy and sustainable school food systems in Vancouver. Canadian Food Studies / La Revue Canadienne des Études Sur l’Alimentation 4(2): 25.

[bibr21-00178969211011522] Ronto R Ball L Pendergast D, et al. (2016) Adolescents’ perspectives on food literacy and its impact on their dietary behaviours. Appetite 107: 549–557. 27614212 10.1016/j.appet.2016.09.006

[bibr22-00178969211011522] Rossi T Kirk D (2020) The pedagogisation of health knowledge and outsourcing of curriculum development: The case of the Stephanie Alexander Kitchen Garden initiative. Discourse: Studies in the Cultural Politics of Education 41(2): 281–298.

[bibr23-00178969211011522] Salam RA Das JK Lassi ZS, et al. (2016) Adolescent health interventions: Conclusions, evidence gaps, and research priorities. Journal of Adolescent Health 59(4): S88–S92. 10.1016/j.jadohealth.2016.05.006 PMC5026678 27664599

[bibr24-00178969211011522] Saxe-Custack A LaChance J Hanna-Attisha M, et al. (2020) Flint Kids Cook: Positive influence of a farmers’ market cooking and nutrition programme on health-related quality of life of US children in a low-income, urban community. Public Health Nutrition 24: 1492–1500. 33028450 10.1017/S136898002000395X PMC10195638

[bibr25-00178969211011522] Shoveller JA Johnson JL (2006) Risky groups, risky behaviour, and risky persons: Dominating discourses on youth sexual health. Critical Public Health 16(1): 47–60.

[bibr26-00178969211011522] Slater J Falkenberg T Rutherford J, et al. (2018) Food literacy competencies: A conceptual framework for youth transitioning to adulthood. International Journal of Consumer Studies 42(5): 547–556.

[bibr27-00178969211011522] Truman E Bischoff M Elliott C (2020) Which literacy for health promotion: Health, food, nutrition or media? Health Promotion International 35(2): 432–444. 30793740 10.1093/heapro/daz007

[bibr28-00178969211011522] Utter J Fay AP Denny S (2017) Child and youth cooking programs: More than good nutrition? Journal of Hunger & Environmental Nutrition 12(4): 554–580.

[bibr29-00178969211011522] Vancouver School Board (VSB) (2010) School gardens. Available at: https://www.vsb.bc.ca/District/Sustainability/garden/Pages/Default.aspx (accessed 16 August 2020).

[bibr30-00178969211011522] World Health Organization (WHO) (1986) The Ottawa Charter for Health Promotion. Copenhagen: WHO, Health Canada, CHPA.

